# A Pilot Study on Emotional Intelligence and Stress Regulation in Hemodialysis Patients

**DOI:** 10.1192/j.eurpsy.2026.11026

**Published:** 2026-07-31

**Authors:** O. Impis, E. Grapsa, A. Zartaloudi, G. Gerogianni

**Affiliations:** 1 Department of Nursing, University of West Attica; 2 Medical School, Professor, University of Athens; 3 Department of Nursing, Associate Professor, University of West Attica, Athens, Greece

## Abstract

**Introduction:**

Emotional intelligence has a close association with management of stress in patients undergoing hemodialysis.

**Objectives:**

This pilot study aimed to assess the associations between emotional intelligence and stress regulation in individuals receiving hemodialysis.

**Methods:**

In this pilot study, forty seven (n=47) patients on hemodialysis completed the questionnaires: i) Wong and Law Emotional Scale - WLEIS) and Trait Emotional Intelligence Questionnaire Short form (TEIQue-SF) for the assessment of emotional intelligence ii) Brief Cope for the assessment of stress management iii) A questionnaire about demographic and clinical characteristics. Spearman correlations coefficients (rho) were used to explore the association of two continuous variables and Mann-Whitney tests for comparison of a continuous variable between two groups. Analyses were conducted using SPSS statistical software (version 26.0).

**Results:**

More use of active positive coping of stress was significantly associated with greater self-control (p=0.014) greater well-being (p=0.032) and greater overall emotional intelligence (p=0.004). More use of humor as a strategy for coping with stress was significantly associated with greater self-control (p=0.034). Substance abuse was significantly and negatively associated with self emotion appraisal (p=0.016) and emotion appraisal of others (p=0.021).

**Conclusions:**

Dialysis patients should attend training programs in order to acquire emotional intelligence skills for an effective stress management.

**Disclosure of Interest:**

None Declared

